# Combining Genetic and Multidimensional Analyses to Identify Interpretive Traits Related to Water Shortage Tolerance as an Indirect Selection Tool for Detecting Genotypes of Drought Tolerance in Wheat Breeding

**DOI:** 10.3390/plants10050931

**Published:** 2021-05-07

**Authors:** Ibrahim Al-Ashkar, Nasser Al-Suhaibani, Kamel Abdella, Mohammed Sallam, Majed Alotaibi, Mahmoud F. Seleiman

**Affiliations:** 1 Plant Production Department, College of Food and Agriculture Sciences, King Saud University, Riyadh 11451, Saudi Arabia; nsuhaib@ksu.edu.sa (N.A.-S.); kkamel@ksu.edu.sa (K.A.); msallam@ksu.edu.sa (M.S.); malotaibia@ksu.edu.sa (M.A.); 2 Agronomy Department, Faculty of Agriculture, Al-Azhar University, Cairo 11651, Egypt; 3 Department of Crop Sciences, Faculty of Agriculture, Menoufia University, Shibin El-Kom 32514, Egypt

**Keywords:** water shortage stress, bread wheat, interpretive traits, agrophysiological traits, genetic parameters, multidimensional analysis

## Abstract

Water shortages have direct adverse effects on wheat productivity and growth worldwide, vertically and horizontally. Productivity may be promoted using water shortage-tolerant wheat genotypes. High-throughput tools have supported plant breeders in increasing the rate of stability of the genetic gain of interpretive traits for wheat productivity through multidimensional technical methods. We used 27 agrophysiological interpretive traits for grain yield (GY) of 25 bread wheat genotypes under water shortage stress conditions for two seasons. Genetic parameters and multidimensional analyses were used to identify genetic and phenotypic variations of the wheat genotypes used, combining these strategies effectively to achieve a balance. Considerable high genotypic variations were observed for 27 traits. Eleven interpretive traits related to GY had combined high heritability (*h*^2^ > 60%) and genetic gain (>20%), compared to GY, which showed moderate values both for heritability (57.60%) and genetic gain (16.89%). It was determined that six out of eleven traits (dry leaf weight (DLW), canopy temperature (CT), relative water content (RWC), flag leaf area (FLA), green leaves area (GLA) and leaf area index (LAI)) loaded the highest onto PC1 and PC2 (with scores of >0.27), and five of them had a positive trend with GY, while the CT trait had a negative correlation determined by principal component analysis (PCA). Genetic parameters and multidimensional analyses (PCA, stepwise regression, and path coefficient) showed that CT, RWC, GLA, and LAI were the most important interpretive traits for GY. Selection based on these four interpretive traits might improve genetic gain for GY in environments that are vulnerable to water shortages. The membership index and clustering analysis based on these four traits were significantly correlated, with some deviation, and classified genotypes into five groups. Highly tolerant, tolerant, intermediate, sensitive and highly sensitive clusters represented six, eight, two, three and six genotypes, respectively. The conclusions drawn from the membership index and clustering analysis, signifying that there were clear separations between the water shortage tolerance groups, were confirmed through discriminant analysis. MANOVA indicated that there were considerable variations between the five water shortage tolerance groups. The tolerated genotypes (DHL02, DHL30, DHL26, Misr1, Pavone-76 and DHL08) can be recommended as interesting new genetic sources for water shortage-tolerant wheat breeding programs.

## 1. Introduction

Water shortages and changes in the world′s climate will lead to an increase in the occurrence of drought phenomena in arid and semiarid areas [1,2,3], and it is important to achieve a horizontal and vertical expansion of field crop production in such areas. These phenomena will be a critically important to achieve sustainable development of crop production, particularly due to farmers rapidly drain groundwater resources through the drilling of deep wells, loss of agricultural lands to urban sustainable development, and demographic pressure. The steady rise in population will, at the same time, continue to require continuous increases in crop production. Improving farming practices and planting drought-tolerant genetic resources are good solutions to sustaining crop production under water deficiency. These solutions aim at greater productivity per valid unit of water than per unit of area [4,5]. Water deficiency is a critical problem that causes abiotic stress and is a nuisance for plant breeders. Therefore, researchers in the relevant fields are seeking credible screening criteria for drought tolerance in wheat genotypes [6].

Plant breeding has proven successful in sustained improvement of drought tolerance in rainfed crops and dry-land crops throughout the years. There are three approaches that might be taken to improve yields with water shortage. The first approach is breeding segregation for yield under water shortage (i.e., yield potential), assuming that this will provide a yield advantage under water shortage conditions. The positive correlation between yield under water shortage and optimal conditions [7,8,9], and indirect selection based on performance under optimal conditions, may not be directly proportional to the selection of drought-tolerant genetic resources [10,11]. The second approach is to breed for better performance of yield under water shortage. However, this is far from ideal, due to the instability of the water shortage constant. In addition, a significant genotype × environment interaction for yield results in a low heritability [6,12]. A third approach is the breeding of tolerance using interpretive yield traits.

Achieving complementarity between physiological and genetic information can provide a more comprehensive genotype model of environment interaction [13]. The interpretive traits to yield offers the best possibility of improving grain yield under water shortage conditions, given that the heritability of grain yield often declines, while the heritability of some interpretive traits continues to be high [14,15]. Interpretive traits were successfully used with a view to better the rate of genetic enhancement for wheat under water shortage conditions [6,16,17]. Several drought screening tests were determined for use in wheat breeding programs [5,17], and the methods for screening for water shortage tolerance for a great number of genotypes should be quick, cost-neutral, stable, and easy to measure [18,19,20]. Some studies are still treating water shortage tolerance as a trait controlled by a single-gene and/or using a visual plant assessment tool, even though water shortage is determined primarily by polygenic inheritance [21,22,23]. Hence, a pyramiding of appropriate morpho-physiological traits was used effectively to evaluating the water shortage tolerance of many crops. The interpretive traits could be evaluated and selected simultaneously in the breeding program. Abdolshahi, et al. [6] introduced a model using agrophysiological traits that were able to anticipate 73 to 80% of yield variation in water shortage environments.

Water shortage increases in plants due to potential losses by evapotranspiration (the aridity index) by preventing the flow of water from soil. Water phase-out from a week to months occurs, depending on the soil characteristics in which plants are cultivated under natural conditions [24]. Water shortages trigger many responses at different levels of plant development, from the molecular to whole-plant level [25], which may include an escape from stress, the capacity for adaptation and tolerance, or averting adverse effects, all of which might occur in parallel [26]. The effect of water stress on photosynthesis can be a direct effect, which increases limitation of the CO_2_-spread pathway via the stomata [27], or an indirect effect, e.g., changes in biochemistry, photosynthetic metabolism, cell membrane permeability [28] and raising oxidative stress [29]. It was determined that gas-exchange measurements are useful indicators of photosynthesis in plants under drought conditions. Leaf mesophyll conductance to CO_2_ may considerably affect genotype performance, and its responses differ from one plant to another, largely because of the differences of genotypic influences on stomatal (stomatal closure) and nonstomatal (diffusional and biochemical) limits of net CO_2_ assimilation rate [28].

It was found that water status measurements are useful indicators of tolerance to water shortage stress in plants [19,20,30], and good tools for indirect selection for tolerance to water shortage stress. These researchers also found a significant correlation of different agrophysiological traits with water shortage stress. A tolerant genotype has the capability of decreasing stress through the conservation of turgid leaves under stress, which has physiological advantages such as growth and stomatal activity, and protects and maintains the photosystem complex [31]. Remotely-sensed infrared canopy temperature (CT) measurements are a cost-effective for rapid, non-destructive monitoring of whole-plant response to water stress [32]. CT relies on the significant inverse association between leaf temperature and transpirational cooling. Genotypes with the capacity to maintain lower CT, transpiration and gas exchange compared to other genotypes under the same stress can be described as tolerant [30,33,34]. Therefore, water status measurements can be used as an indirect selection criterion for tolerance to water shortage stress in wheat breeding. The breeding of water shortage-tolerant genotypes to yield is the main goal of plant breeders. Interpretive traits are plant attributes associated with final grain yield (GY) under water shortage conditions, and may provide a greater amount of information for plant breeders for the selection process of drought-tolerant genotypes [35]. There is now a crucial need to improve the comprehensive understanding of an indirect approach, and the mechanisms of deep-rooted adaptive behavior, based on incorporating multiple morphophysiological plant traits associated with drought tolerance with yield and its components. This can assist in targeting major traits that may enhance genetic gain for grain yield in drought-prone areas [5,11].

The inaccurate depiction of interpretive (physiological, morphological and destructive) traits related to water shortage stress, and low genetic variation of available wheat varieties, is currently one of the leading causes for limited success when breeding drought-tolerant wheat varieties [19,36]. Breeding improvement strategies for drought-tolerant genotypes rely on the use of multidimensional methods to obtain a more reliable assessment of a great number of genotypes (varieties and lines) and traits. These strategies include an integrative approach that combines various parameters of physiological traits at the level of leaves, and of whole-plant, agronomic traits [6,37,38]. Providing useful information for plant breeders to determine the adaptive conduct of plants under water shortage stress requires the use of a combination of reliable phenotypic traits and high-powered computer modelling of multidimensional data to provide a greater understanding of the complicated mechanisms that function under abiotic stress [6,12,34,36,37]. Therefore, screening tests are required for statistical analysis with the capacity to precisely explain tolerant and sensitive genotypes under water shortage. Therefore, multivariate analysis techniques (e.g., multicollinearity, multiple regression, principal component analysis, path analysis, MANOVA and discriminant analysis) could serve as a useful instrument to identify sources of variation in water shortage-tolerance using precise, reliable and multiple selection criteria [5,6,19].

The principal aim of this study was to describe the characteristics of double haploid wheat lines compared to numerous cultivars in response to water shortage stress, and to identify interpretive traits associated with water shortage tolerance based on their heritability, genetic gain and multidimensional evaluation. Another aim was to identify genotypes with higher water shortage tolerance that can be utilized to breed water shortage-tolerant varieties.

## 2. Material and Methods

### 2.1. Plants and Experimental Design

A set of 25 genotypes (seven varieties and 18 lines) of bread wheat were used in this study (Table S1). The varieties were chosen from different ecological regions based on the presence of broad genetic differences between them with respect to drought tolerance and drought sensitivity. Five of them were provided by the Agricultural Research Center, Egypt. Seeds of the other two, KSU 106 and Pavone-76, were collected from College of Food and Agriculture Sciences, King Saud University, Riyadh, Saudi Arabia. The 18 double haploid lines (DHLs) were provided through the Agronomy Department, Faculty of Agriculture, Al-Azhar University, Nasr City, Cairo, Egypt, and from published literature [39]. All genotypes were sown in two consecutive seasons (2018/19; S1 and 2019/20; S2) as five-row (3.0 m long) plots, with a distance between rows of 0.17 m, at the King Saud University Agricultural Research Station (24°42′ N, 44°46′ E, 400 m asl) in a split-plot design with three replications. Seedling rate was 360 germinating kernels m^−2^, and the fertilizing rates used were 1.2 kg m^−2^ N and 0.8 kg m^−2^ P_2_O_5_. During the growing season, the temperature and rain were 12.9–32.2 °C, and 8.0–25.0 mm, respectively. The soil texture was classified by electrical conductivity as sandy loam (2.89 dS m^−1^). Two irrigation regimes were used two weeks after sowing. The first regime (full irrigation) was watered to 100% field capacity upon reaching a cumulative evaporation of 50 mm, and the second regime (limited irrigation) was watered to 33% field capacity upon reaching a cumulative evaporation of 150 mm. The main plots were allocated to two irrigation regimes, while the subplots were allocated to the genotypes.

### 2.2. Measurements of Morpho-Physiological and Agronomic Traits and Data Collection

#### 2.2.1. Leaf Water Status Parameters

Leaf water status was measured by canopy temperature (CT), leaf water content (LWC), relative water content (RWC) and leaf equivalent water thickness (LEWT) at the flowering stage on the flag leaf. CT was measured under a cloudless sky before noon (11:30 to 12:00 h) using an infrared thermometer (Therma CAM SC 3000 infrared camera, FLIR System Inc., North Billerica, MA, USA). The images were captured at a height of approximately 1 m over the top of the ear and converted into CT using the FLIR Quick Report 1.2 software package. LWC and RWC were measured for five leaves collected randomly from each genotype, and then weighed directly to capture data related to fresh weight (FW). The same leaves were flooded with water at 25 °C until they were fully turgid, then dehydrated with tissue paper to remove any excess water, and weighed to capture data on turgid weight (TW). Finally, the same leaves were oven-dried for 48 h at 70 °C and weighed to capture data on dry weight (DW). Data taken from FW, TW and DW were used to calculate LWC, RW, and LEWT, according to the following equations [40,41,42]:

(1) LWC = FW − DW/FW × 100

(2) RWC = FW − DW/TW − DW × 100

(3) LEWT = FW − DW/Flag leaf area

#### 2.2.2. Photosynthetic Parameters

Photosynthetic parameters were measured in the grain-filling stage using a Li-6400 gas exchange system (Li-Cor, Inc., Lincoln, Nebraska, USA), starting at 10:00 AM until 12 noon. The upper third of the flag leaves was used. Photosynthesis rate (Pn), transpiration rate (E), stomatal conductance (Gs), intracellular CO_2_ concentration (Ci), and atmospheric CO_2_ concentration (Ca) were measured at a saturating photosynthetic photon flux density of 700 μmol photons m^−2^ s^−1^. Temperature, CO_2_ concentration, and relative humidity were measured at 26 ± 2 °C, 485 ± 23 μmol/L and 65 ± 7%, respectively. Instantaneous water use efficiency (WUE), intrinsic water use efficiency (WUEi), and stomatal limitation value (Ls) were calculated using data taken from Pn, E, Gs, Ci and Ca, according to the following equations [43]:

(4) WUE = Pn/E

(5) WUEi = Pn/Gs

(6) Ls = 1 − Ci/Ca

#### 2.2.3. Morphological Parameters

Five plants from the middle rows of each pilot module were randomly selected to calculate green leaf number (GLN), flag leaf area (FLA), and green leaf area (GLA) at the mid anthesis stage. All green leaves were removed from the five plants and run through an area meter (LI 3100; LI-COR Inc., Lincoln, NE, USA) to calculate surface GLA. Thereafter, all parts of the five plants (stem, green and complete brown leaves, sheath parts and spike) were oven-dried at 70 °C for 72 h to a constant weight and then weighed to obtain dry stem weight (DSW), dry leaf weight (DLW), and total dry weight (TDW). Data taken from GLA (A), number of plants in 1 m (B), number of plants used (C = 5, in this study), and distance between rows (D = 17 cm, in this study) were used to calculate leaf area index (LAI), according to this equation [5]:

(7) LAI = (A × B)/(C × D)

#### 2.2.4. Agronomic and Yield Traits Measurements

The middle rows were used to lower the environmental impact in each plot for measuring agronomic and yield traits. Days to heading (DH, days) were recorded when 50% of plants had headed and flowered, and days to maturity (DM, days) were recorded when the tops of the peduncles of 50% of plants were yellow. Grain filling duration (GFD, days) was calculated during the period from DM and DH. Plant height (PH, cm plant^−1^) and spike length (SL, cm) were measured in 10 plants chosen randomly from each plot after excluding awns and upon reaching maturity. The number of spikes (NS, m^−2^), and number of spikelets (NSS, spike^−1^) were counted at the same time. The plants were threshed to measure the number of kernels (NKS, spike^−1^), thousand-kernel weight (HKW, g), and grain yield (GY, ton ha^−1^). Three rows (2 m long) were used to estimate GY.

#### 2.2.5. Statistical Analysis

Analysis of variance for all traits was performed using the SAS v9.2 software package (SAS Institute, Inc., Cary, NC, USA) for each individual season with full and limited irrigation. A combined analysis was performed across the two seasons to test the homogeneity of error variance assuming season is a random effect, and genotypes and irrigation regimes are both fixed effects. The results indicated homogenous variance across two seasons for all measured traits, and based on this test the data of the two seasons were combined and analyzed according to Gomez and Gomez [44]. Subsequently, the mean squared values of genotype, genotype × environment, error and replicates were used to calculate the variance components following the methods proposed by Fehr [45] and described by Al-Ashkar, et al. [37], which allowed for the calculation of genetic parameters for all traits as follows:

(8) $$\mathrm{Heritability}\;\left(h^{2},\;\mathrm{broad}\;\mathrm{sense}\right)=\left(\sigma_{g}^{2}\right)/\;\left(\sigma_{g}^{2}+\frac{\sigma_{g\times e}^{2}}{e}+\frac{\sigma_{\mathrm{re}}^{2}}{r\times e}\right)$$

(9) $$\mathrm{Genetic}\;\mathrm{advance}\;(\mathrm{GA})=\frac{\sigma_{g}^{2}}{\sigma_{p}^{2}}\;\times \;\sqrt{\sigma^{2}g}\;\times \;k$$

(10) $$\mathrm{Genetic}\;\mathrm{gain}\;(\%)=\frac{\mathrm{GA}}{\overset{\text{¯}}{X}}\times 100$$

(11) $$\mathrm{Genotypic}\;\mathrm{coefficient}\;\mathrm{of}\;\mathrm{variability}\;(\mathrm{GCV})=\frac{{\sqrt{\sigma }}_{g\;}^{2}}{\overset{\text{¯}}{X}}\times 100$$

(12) $$\mathrm{Phenotypic}\;\mathrm{coefficient}\;\mathrm{of}\;\mathrm{variability}\;(\mathrm{PCV})=\frac{{\sqrt{\sigma }}_{p\;}^{2}}{\overset{\text{¯}}{X}}\times 100$$

where $\sigma_{g}^{2}$ is the genotypic variance, $\sigma_{P}^{2}$ is the phenotypic variance, $\sigma_{g\times e}^{2}$ is the genotype × environment variance, $\sigma_{\mathrm{re}}^{2}$ is the residual variance (error), r is the number of repetitions, e is the number of environments, $\overset{\text{¯}}{X}$ is the overall mean for each trait and k is the selection differential (and value of 2.06) at 5% selection intensity.

The multicollinearity test was used to uncover multicollinearity sources in a correlation matrix of interpretive traits to their exclusion. Multidimensional modeling was used to better understand correlations between a large number of measured traits, their Path coefficient analysis to partition variation into multiple direct and indirect effects, depending on SMLR output and path coefficient analysis and a membership index, was used to characterize the drought tolerance index (DTI) values of all tested genotype contributions and their encapsulation into major components. Principal component analysis (PCA) was performed, based on data provided by the correlation matrix, to reduce the dimensions of the data space and reliance on the first two components of PCA. Stepwise multiple linear regression analysis (SMLRA) was used to determine the most powerful interpretive traits that contribute to the most variability in the intrigued variable (GY), depending on the output of PCA. The DTI values of the genotypes were calculated using four influential interpretive traits (LAI, RWC, CT and GLA), and the membership index was used in the computation [46]. The membership index value $\left(F_{\mathrm{ij}}\right)$ was counted to every interpretive trait per genotype as the ratio of limited irrigation values to full irrigation values per year. The overall mean of the membership index of these the traits ($F_{i}$) was used as the tolerance index to water shortage stress.

(13) $${\;F}_{\mathrm{ij}}=\frac{x_{\mathrm{ij}\;-}{\;x}_{\min}}{x_{\max\;-}{\;x}_{\min}}\;\mathrm{and}\;F_{i}=\mathrm{average}\;\mathrm{of}\;F_{\mathrm{ij}\;}$$

where $x_{\mathrm{ij}\;}$ is the ratio of the *i*th genotype, *j*th trait, and $x_{\min}$ and $x_{\max\;}$ are the minimum and maximum ratios of the trait. All genotypes were classified into five ranks, as being highly tolerant (HT, Rank 1: F_i_ > 0.8), tolerant (T, Rank 2: 0.6 ≤ F_i_ *<* 0.8), moderately tolerant (MT, Rank 3: 0.4 ≤ F_i_ < 0.6), sensitive (S, Rank 4: 0.2 ≤ F_i_ < 0.4), and highly sensitive (HS, Rank 5: F_i_ < 0.2).

The membership index value (score) used in the calculation of the genetic similarity matrix (cluster analysis) between genotypes from three interpretive traits was based on the Euclidean distance dissimilarity coefficient using UPGMA (Unweighted Pair Group Method with Arithmetic Mean). The matrix of Euclidean distance dissimilarity coefficient was used in the calculation of the principal coordinate analysis (PCoA) to reduce the dimensions of data space. Discriminant function analysis (DFA) and MANOVA used the same data as the DTI score per genotype (the four traits as quantitative variables, with the five classes HT, T, MT, S, and HS as qualitative variables) in order to confirm the categorization of genotypes. All genotypes were then given an equal prior probability to be classified into the five classes of drought tolerance. Statistical analysis (Multicollinearity test, PCA, SMLRA, cluster analysis, PCoA, DFA and MANOVA) was carried out using the XLSTAT statistical software package (vers. 2019.1, Excel Add-ins soft SARL, New York, NY, USA).

## 3. Results

### 3.1. Phenotypic Variability of Measured Traits Across Seasons and Genotypes

All measured traits presented highly significant differences (*p* < 0.01) for treatments (irrigation levels and genotypes) and their interaction in each season, based on ANOVA. The only exception was interaction of DH in S1, irrigation levels of NKS in S2, and levels of irrigation of Pn in S1 and S2, being nonsignificant (Table S2). Across the two seasons all interactions were significant for NS, PH, FLA, GLA, LAI, NSS, RWC, HKW, CT, LEWT, GS, WUE and WUEi traits, and nonsignificant for DH, DM, GFD, Pn, SDW, DLW, and TDW traits. Interaction (seasons × levels irrigation) were nonsignificant for GLN, SL, LWC, Ci, E and LS (Table 1).

Interestingly, in each and across the two seasons, there was great variation between the lowest value and the highest value for the measured traits, with certain exceptions for some traits, which presented a narrow variation between the lowest value and the highest value. The maximum values were nearly two times higher than the minimum values for most measured traits (Table 2), indicating high genetic diversity between genotypes used. Most measured trait values decreased under limited irrigation compared to full irrigation, with the exception of traits CT, WUE, WUEi, and LS, which showed the opposite direction, as shown in Table 2.

### 3.2. Estimation of Genetic Parameters for Measured Traits

The results showed that the five genetic parameters (i.e., heritability (*h*^2^), genotypic (GCV), and phenotypic coefficient of variability (PCV), genetic advance (GA), and genetic gain (GG)) varied greatly for all measured traits. The *h*^2^ demonstrated a wide range between measured traits, which varied from 29.99% for WUEi to 97.82% for DH. The ratio of PCV to GCV was proximate for some traits and the GCV was smaller than the PCV, except for ten traits (NS, LAI, SL, NSS, LWC, GY, Gs, Ci, E, WUEi, and LS), which showed a greater percentage of PCV to GCV. Genetic advance (GA) and genetic gain (GG) showed high diversity for all measured traits (Table 3). GA and GG ranged from 0.01 (LEWT) to 90.46 (NS) and from 3.14 (LWC) to 51.71% (LEWT), respectively (Table 3).

### 3.3. Multicolinearity and Principal Component Analysis

**Figure 1:**
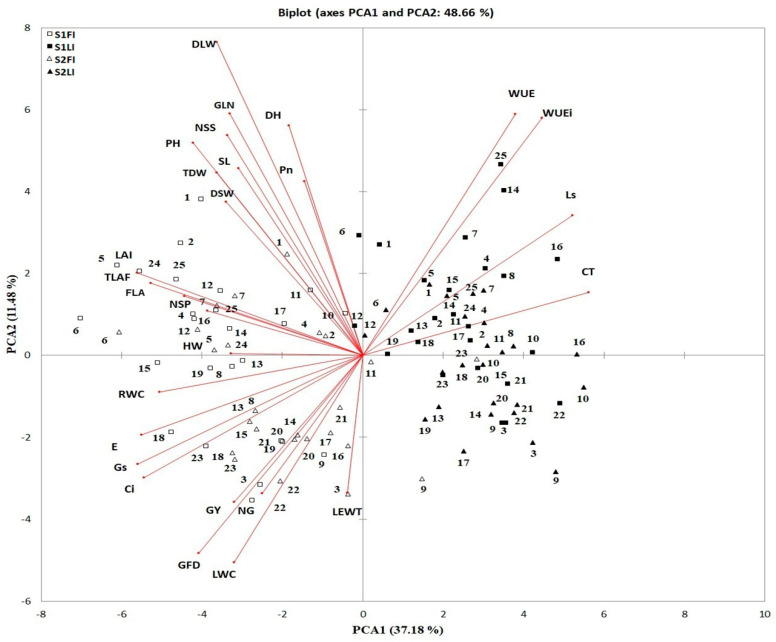
Biplot for the first two principal components in the principal component analysis (PCA) for 27 measured traits of 25 wheat genotypes in two seasons (S1 and S2) under full (FI) and limited irrigation regimes (LI). The numbers were indicated according to Table 6. Leaf water content (LWC), relative water content (RWC), canopy temperature (CT), leaf equivalent water thickness (LEWT), photosynthesis rate (Pn), stomatal conductance (Gs), intracellular CO_2_ concentration (Ci), transpiration rate (E), instantaneous water use efficiency (WUE), intrinsic water use efficiency (WUEi), stomatal limitation value (Ls), green leaf number (GLN), flag leaf area (FLA), and green leaf area (GLA), leaf area index (LAI), dry stem weight (DSW), dry leaf weight (DLW), total dry weight (TDW), days to heading (DH), grain filling duration (GFD), plant height (PH), spike length.

The results showed that the multicolinearity analysis varied greatly between measured traits. The tolerance (T) and variance inflation factors (VIF) ranged from 0.00 to 0.465 and from 0.00 to 10.00, respectively, except for three traits (DH, DM and GFD), which showed VIF values >10.0, before excluding traits (Table 4). After excluding the DM trait (>multicolinearity), T and VIF ranged from 0.00 to 0.470 and from 0.00 to 9.62, respectively. A PCA was conducted for each of the 27 measured traits, 25 genotypes and two irrigation treatments in two seasons simultaneously (Figure 1). The first six principal components had eigenvalues greater than 1, which explained 78.09% of the total variation (Table S3). The first two PCAs explained 37.18 and 11.48% of the total phenotypic variation between 27 traits, respectively, and 17 traits of them loaded the highest onto PC1 and PC2 (with scores of >0.27). PCA resulted in a clear separation between irrigation treatments and genotypes based on measured traits to identify the main trait, which could be used in identifying traits that explained much of the variation between 25 wheat genotypes used. In PC1, the four traits (CT, Ls, WUE and WUEi) were combined in a positive trend, and the 23 other traits were spread in a negative trend. In PC2, 18 traits were combined in a positive trend and the nine other traits were spread in a negative trend. More importantly, the angle between the vectors of traits was acute (less than 90°) for GY with most traits, which indicates positive correlation, while the angle between the vector of the four traits (CT, Ls, WUE and WUEi) was higher than 90° for GY, which indicated negative correlation (Figure 1).

### 3.4. Identification of Traits Related to Yield Performance

The relationships between all traits suggested to be very important by PC1 and PC2 were analyzed using SMLR and path coefficient analysis in order to understand the best-measured and yield-related traits and their contribution to yield performance (Table 5). The results of SMLR showed that GLA, LAI, RWC, CT and Gs were significantly correlated to GY, and their contribution rates were 0.104, 0.141, 0.171, 0.400 and 0.070, respectively (Table 5). The R^2^ of SMLR model was 0.886. The five components of GY variation partitioned into multiple direct and indirect effects using path coefficient analysis. Each direct and indirect effect contributed to 0.565 (CT, alone possessed 0.426 of them) and 0.321, respectively. The R^2^ value was the same as that in the SMLR model (0.886), with a noise value of 0.338. It was concluded that these traits (GLA, LAI, RWC, CT and Gs) may be relevant and important selection criteria and can be relied on in defining the levels of drought-tolerant and drought-sensitive genotypes of wheat, coupled with high correlation with yield.

### 3.5. Classification of Drought-Tolerance of Twenty-Five Wheat Genotypes

The four traits (GLA, LAI, RWC and CT) were used in classification of tested genotypes after the Gs trait was removed owing its low-level heritability and genetic gain. We used these four traits in finding a membership index for the phenotypic profiling of 25 wheat genotypes to assess the extent of their drought-tolerance. The membership index was obtained by scores calculated from these four traits into five main clusters (Table 6). Regardless of growing season and the four traits used, the genotypes were classified in as follows. The first group was classified as highly tolerant (HT, with the highest score of F*i* > 0.8), and included eight genotypes (DHL02, DHL30, DHL26, Gemmeiza-9, Misr1, DHL05, Pavone-76 and DHL08). The second group was classified as tolerant (T, with scores of 0.6 ≥ F_i_ < 0.8), and included six genotypes (DHL12, DHL25, DHL07, DHL01, Giza-168 and DHL03). The third group was classified as moderately tolerant (I, with scores of 0.4 ≥ F_i_ < 0.6), and contained four genotypes (DHL11, Gemmeiza-9, DHL29 and DHL23). The fourth group was classified as sensitive (S, with scores of 0.2 ≥ F_i_ < 0.4), and contained seven genotypes (KSU106, DHL14, DHL15, DHL06, Sakha-93, DHL21 and DHL22). In each growing season (S1 and S2) separately, 20 out of 25 genotypes were similarly categorized from their combined scores. Some genotypes in the fourth group were classified as highly sensitive (HS, with scores of F_i_ < 0.2) with some traits and/or season combinations such as DHL21 with LAI in S1, DHL14 with LAI in S2 and DHL06 with CT in S2 (fifth group).

### 3.6. Clustering and Genetic Relationships between the Genotypes for Drought Tolerance

**Figure 2:**
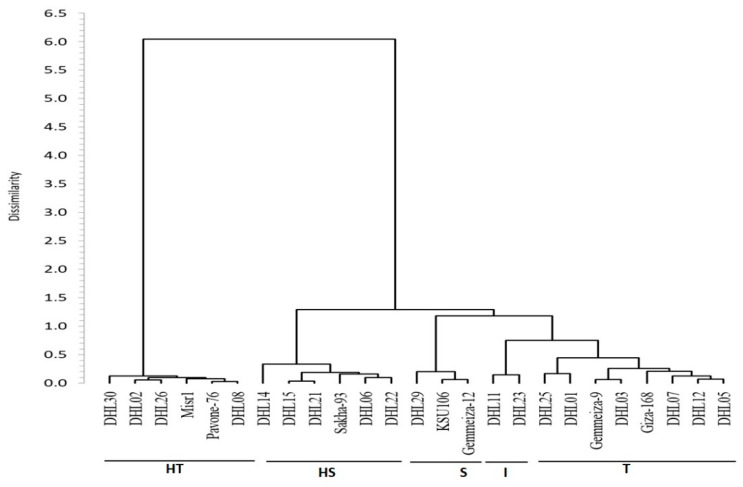
Dendrogram showing clustering of 25 wheat genotypes based on the Euclidean distance of relative water content, canopy temperature, leaf area index and green leaf area.

We decided to use the membership index scores of the four traits (GLA, LAI, RWC and CT) to create a cluster analysis for the phenotypic profiling for drought tolerance in the wheat genotypes. The clustering was the genetics dissimilarity matrix based on Euclidean distance using Ward’s method of agglomeration. The genotypes produced five major clusters, clearly separated with a dissimilarity coefficient of 0.599 and reflected the distance between the five clusters based on the tolerance or sensitivity of wheat genotypes for drought. The highly tolerant, tolerant, intermediate, sensitive and highly sensitive clusters represented six, eight, two, three and six genotypes, respectively (Figure 2).

**Figure 3:**
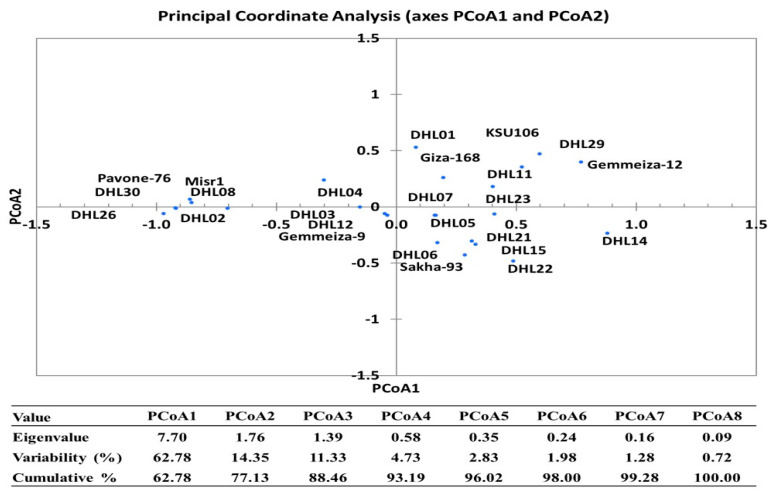
Principal coordinates analysis (PCoA) among the 25 wheat genotypes based on the Euclidean distance dissimilarity.

The classification relationships based on membership index and clustering of drought-tolerant and sensitive genotypes were significantly correlated (r = 0.328, *p* < 0.0001) according to the Mantel test. The first three PCoAs (two-dimensional) had eigenvalues greater than 1, which explained 88.46% of the total variation (PCoA1 and PCoA2 explained 62.78% and 14.35%, respectively). PCoA resulted in a clear separation between genotypes groups based on tolerance or sensitivity for drought. S and HS groups were distributed into quadrants 1 and 2, respectively, but the I group was distributed in both quadrants (Figure 3). Group T was the largest, distributed in all quadrants and covered the largest PCoA area. The HT group was distributed into quadrants three and four. The genotypes groups (into PCoA) were in full conformity with results from clustering analysis.

### 3.7. Differentiation of Drought Groups by Discriminant Function Analysis and MANOVA

**Figure 4:**
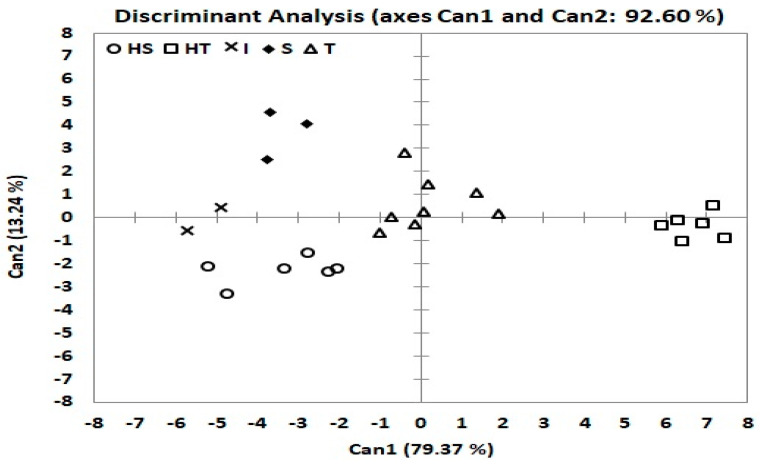
Distribution of 25 wheat genotypes by canonical discriminant analysis (Can) of relative water content, canopy temperature, leaf area index and green leaves area traits responses to drought stress. Highly tolerant (HT), tolerant (T), intermediate (I), sensitive (S) and highly sensitive (HS).

Fisher linear discriminant analysis (FLDA) operates in a manner similar to MANOVA, which initially computes the Mahalanobis distance of each genotype to a group and then uses this distance to categorize a genotype into the group with the smallest generalized squared distance [47]. The homogeneity test was significant for covariance matrices (0.04 < *p* < 0.0001), so we were prompted to use quadratic discriminant analysis (QDA). Its results had a 0.00% error rate, confirming that the classification of our genotypes using clustering based on membership index was an influential analysis. Discriminant analysis was used to better understand the grouping and evaluate the extent of the differences between drought groups. The four selected measures (GLA, LAI, RWC and CT) were high and significant with each statistical multivariate analysis used, thus confirming the odds of prediction by clustering based on membership. The discriminant functions (two-dimensional) of five groups and four selected measures were closely associated for the prediction of membership into drought groupings for the 25 genotypes used (Figure 4). The first three canonical discriminant functions (Can) explained 79.37, 13.24, and 6.17% (total of 98,77) of the total phenotypic variations in the four traits (which had eigenvalues greater than 1), respectively (Table S4).

Loading the four variables in S1 and S2 to canonical discriminant functions showed that GLA, LAI, RWC and CT were positive and highly correlated to Can1 (Table S4). In addition to the variance explained by Can1, it appeared that Can1 is a measure of the overall characteristics of drought tolerance by the four measures. Can2 was closely interrelated to GLA and RWC, but negatively correlated to LAI and CT in each of S1 and S2. Can3 was closely interrelated to GLA and LAI, but negatively correlated to RWC and CT in each of S1 and S2. Therefore, this result suggests that Can2 differentiates genotypes based on GLA and RWC, while Can3 differentiates genotypes based on GLA and LAI. The maximum separation of group means was observed between HT and I (6.67 vs. −5.04), and the separation between S and HS was very close (−3.41 vs. −3.48) in Can1. The maximum separation between group means was observed in S vs. HS (3.70 vs. −2.03) in Can2. Examination of Can3 showed a maximum separation of HS from group I (1.12 vs. −3.40). Two (HT, and T) groups with positive values to Can1, which had some tolerance to drought and, conversely, groups HS, I and S, had negative values to Can1. In the plot of drought groups with Can1 and Can2, group I was placed in the middle between groups HS and S (Figure 4). Group HT had a positive value to Can1 (6.67) and negative values to Can2 and Can3 (−0.35 and −0.06), indicating that HT had high mean values in all traits, but negative low LAI and CT in Can2, and RWC, and CT in Can3. The S group was against the HT group, which had a negative value to Can1 (−3.41) and positive value to Can2 and Can3 (3.70 and 0.79). The I group had negative values to three Can (−5.035, −0.867, and −3.401), respectively (Table S4).

### 3.8. Phenotypic Variation Among Drought Groups

**Figure 5:**
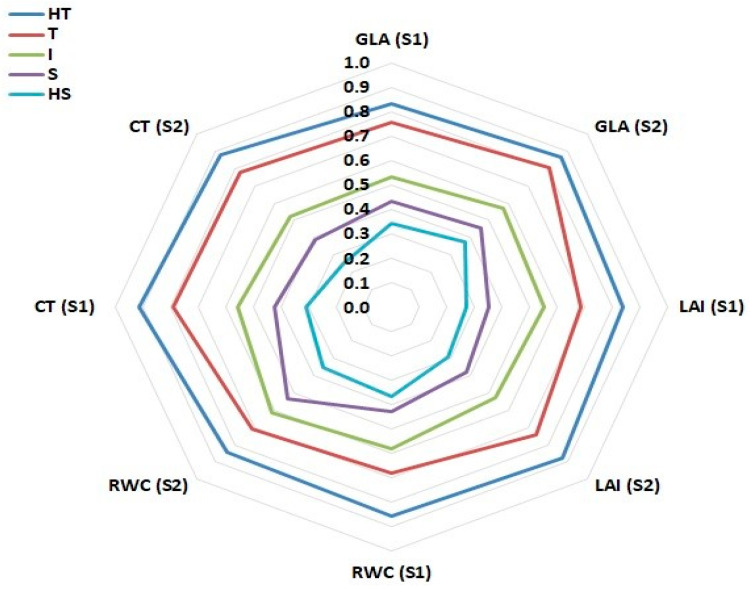
Radar charts comparing four traits of the five groups. Data were analyzed using least squares (LS) in two seasons. Relative water content (RWC), canopy temperature (CT), leaf area index (LAI), and green leaf area (GLA).

Multivariate analysis of variance (MANOVA) indicated that the five groups were significantly different for four traits in S1 and S2 (Figure 5). This also indicated the complete separation between the five groups based on the four quantitative traits in S1 and S2. The LS means comparison for each trait between groups showed significant differences in HT with the T, I, S and HS groups in all traits for S1 and S2, though group T in S2 was insignificant for the GLA trait (Table S5). Group T exhibited significant differences from the I, S and HS groups in all traits in S1 and S2. Group I exhibited significant differences from the S and HS groups in all traits in S1 and S2, except for group S in S2 which was insignificant for the RWC trait. Group S vs HS exhibited significant differences in all traits in S1 and S2, except for the RWC trait, which was insignificant in S1.

## 4. Discussion

Plant breeders rely on several interpretive traits as screening criteria to evaluate the water shortage tolerance of genotypes and increasing access to sustainable genotypes appropriate for water shortage [48,49]. Interpretive traits, such as agronomic traits (yield components, leaf water status, photosynthetic and morphological measures) reflect the integration of many plant operations either on the whole plant or at distinct stages of the life-cycle. Above-ground biomass (TDW), canopy temperature (CT), leaf area index (LAI), and green leaf area (GLA) reflect radiation use efficiency, plant competition, photosynthesis and evapotranspiration rates, and the status of crop growth under special growing conditions [5,6,50], and are thus important traits. Stomatal conductance (Gs) reflects the relationship between photosynthesis and transpiration capacity and is influenced by the close relationship between external and/or internal environmental conditions and plant characteristics induced by water stress [51,52]. The overall response of plant tissues to soil water shortages is a signal of shortages in relative water content (RWC), which depends on the extent and duration of the water shortage stress [53].

The accurate selection of interpretive traits is efficient if these traits are closely linked with GY and have high-value heritability and genetic gain [20,54,55]. There were highly significant differences (*p* < 0.01) between treatments and their interactions (except for some traits, which were nonsignificant in one of the interactions (Table 1), based on ANOVA. Specifically, there was great variation between the lowest and highest values for most measured traits, indicating the high genetic diversity between the genotypes used. The interactions in ANOVA between the three treatments (genotypes, irrigation and seasons) were significant with most traits, suggesting that the performance of the genotypes differed from one level of irrigation to another and one season to another (Table S2).

There were eleven (SDW, DLW, TDW, FLA, GLA, LAI, RWC, NKS, CT, LEWT, and Pn) traits with high heritability (*h*^2^ > 60%) and genetic gain (>20%), coupled with their approximate values for GCV and PCV (Table 3). The combination of high *h*^2^ (<60.0%), genetic gain (>20.0%), and GCV for the trait, evidenced that the variation between genotypes was largely due to the additive genetic part. The selection process had a large confidence interval with these traits [5,6,54]. The results showed that grain yield had moderate values both for heritability (57.60%) and genetic gain (16.89%), which would slow the direct selection progress of breeding programs under water shortage and its considerable environmental interaction (Table 1 and Table 3). The findings of these genetic analyses for interpretive traits suggest that accurate identification of water shortage tolerance genotypes can be improved, compared to identification based on measuring grain yield only under water shortage stress. This is because stress reduced the heritability of grain yield under water shortage, which remained high for some interpretive traits [6,14,37]. Furthermore, a selection process based on interpretive traits is expected to be efficient at an early growth phase, where it is not likely to affect grain yield. Moreover, SDW, DLW, TDW, FLA, GLA, LAI, RWC, NKS, CT, LEWT and Pn had both higher heritability and genetic gain than GY (Table 3). These results reinforce the idea of breeding for interpretive traits to high yield genotypes under water shortage stress conditions [6]. Therefore, the interpretive traits, which have a high value of both high heritability and genetic gain combined, can be used as precise and reliable screening criteria for evaluating the genotypes for water shortage tolerance [37,56,57], particularly if the measurement method is easy, quick and low-cost [19].

The multicolinearity test has been used to determine if multicollinearity exists between measured traits, and when there is multicollinearity to take appropriate measures to adjust it [58,59]. The results showed that three traits (DH, DM, and GFD) had VIF values >10.0, and after excluding the DM trait (>multicollinearity), all VIF values of <10.0. After excluding the DM trait, PCA was used for identifying the most significant measured traits with three treatments (genotypes, irrigation and seasons), owing to the significant interactions seen by ANOVA (Table 4). The first two PCA explained 37.18% and 11.48% of the total phenotypic variation between 27 traits, respectively, and 17 traits loaded the highest onto PC1 and PC2 (with scores of >0.27) and were seen as paramount (Table S3). PCA resulted in a clear separation between irrigation treatments and genotypes based on measured traits to identify the critical trait. This was used for identifying traits that clarified much of the difference between the 25 wheat genotypes used. The angle between the vectors of traits was acute (less than 90°) for GY with most traits, which indicates that they are positively correlated, while the angle between the vector of the four traits (CT, Ls, WUE and WUEi) was higher than 90° for GY, which indicates that they are negatively correlated (Figure 2). The 16 interpretive traits (GFD, DLW, NS, PH, FLA, GLA, LAI, NSS, RWC, CT, Gs, Ci, E, WUE, WUEi and Ls) in PC1 and PC2 (with scores of >0.27) were used as influential screening criteria for yield (Supplementary Table S3, Figure 1) and were used in SMLR and path coefficient analysis.

Multivariate analyses (SMLR and path coefficient) are effective instruments for understanding the relationship between interpretive traits for yield [60,61]. It has also been found that using simple correlation without regard to interactions between interpretive traits for yield may not be useful for finding successful breeding programs [62]. The ineffective impact from the 16 selected interpretive traits from PCA analysis on yield was removed by SMLR. In this study, CT, RWC, LAI, GLA and Gs, according to the order of their importance, were found to be credible interpretive traits for GY (*p* < 0.01, Table 5). SMLR had a coefficient of determination (R^2^) of 0.886. Many investigators have used multivariate analyses [61,63,64]. Based on SMLR, we used path analysis to separate the five interpretive traits obtained by SMLR into the direct and indirect impacts for each trait. If the correlation between interpretive traits and yield is due to a direct effect, it suggests a relationship between them and they are selected to improve performance [61,65]. The separation of the correlation values into direct and indirect impacts was close for LAI and RWC. However, for CT, the direct impact was much greater than the indirect impact, while the opposite was true for GLA and Gs (Table 5). The separations of the coefficient of determination for direct and indirect impacts were 0.565 and 0.321, respectively, and most of the direct impact was due to a contributing to the CT trait. Combining genetic analyses (heritability and genetic gain) and multivariate analyses (PCA, SMLR and path coefficient) excluded the Gs interpretive trait due to its reduced heritability and genetic gain, which was 30.37 and 9.69, respectively. Hence, we concluded that LAI, RWC, CT and GLA are good interpretive traits for predicting yield and could be unbiased traits to evaluate the genotypes for water shortage tolerance in view of their important contribution to grain yield productivity (Table 5). It would be normal for genotype performance to differ from one trait to another, but at least it will be superior in one trait [61].

CT and RWC are useful indicators of water status in plants [30,53], and leaf area index (LAI), and green leaf area (GLA) reflect the overall situation of growth, radiation use efficiency, plant competition, photosynthesis and evapotranspiration rates [5,50]. CT is a powerful tool for the indirect selection of genotypes for water shortage tolerance, given the interdependencies with several morphophysiological (photosynthetic capacity and chlorophyll content) and agronomic traits (yield and yield component) under water shortage stress, and several traits connected with water status in plants [66,67,68]. Genotypes that have the capability to lower CT and gas exchange are more desirable, because they have greater efficiency of transpiration and gas exchange as leaf-cooling responses under water shortage stress [30,33]. Therefore, CT has been used in wheat breeding programs as a powerful selection tool for stress tolerance. CT varies from genotype to genotype and this may be due to differences in the plant′s ability to move water across the vascular system by regulating stomata aperture that drives transpiration and affects metabolism, root biomass, root depth and source sink balance [69]. As such, CT is considered a powerful physiological trait and considered a cost-effective nondestructive measure for identifying water shortage tolerant genotypes [17,66,67]. The focus is on finding genotypes that preserve lower CT in plant breeding programs and selecting water shortage-tolerant varieties, as in our study. Membership indices in the first two groups were HT and T (with the highest score), and included eight genotypes (DHL02, DHL30, DHL26, Gemmeiza-9, Misr1, DHL05, Pavone-76 and DHL08) and six genotypes (DHL12, DHL25, DHL07, DHL01, Giza-168 and DHL03), respectively, compared to the fourth and fifth groups (S and HS), which were in the reverse direction under the same conditions (Table 6).

RWC is a robust mechanism to preserve cellular hydration by osmotic adjustment as a barometer of plant water status. Trials can be rapidly performed to identify genotypes that preserve high leaf RWC values during water deficit stress. Tolerant genotypes have the potential to minimize stress by keeping leaves turgid under water deficit stress through their possession of certain physiological advantages, which protect and preserve the photosystem complex, plant growth and stomatal activity [31]. The low value for the membership index of RWC indicated that the photosynthetic ability of the sensitive genotypes under water deficit stress was limited due to lack of water and cellular dehydration [53,70]. The membership index was highest in groups HT and T, and included 14 genotypes, compared to groups S and HS, which included seven genotypes. Genotype groups HT and T were less affected by water deficit stress due to biochemical activities which prevent oxidative damage by multiple mechanisms (photosynthesis, heat fragmentation by xanthophyll pigments, and electron transfer to oxygen acceptors other than water), which might relate to differences in the closure level of the stomata and/or responses that enhance CO_2_ fixation [70].

The clustering analysis results, based on a dissimilarity matrix and Euclidean distance using Ward’s method of agglomeration, showed that all genotypes were assigned to five groups (clusters) based on phenotypic profiling for drought tolerance (Figure 2). The five groups were clearly separated and reflected the distance between the five clusters based on the tolerance and/or sensitivity of wheat genotypes for drought. The highly tolerant, tolerant, intermediate, sensitive, and highly-sensitive clusters represented six, eight, two, three and six genotypes, respectively. The groups showed some deviation to the nearest group, compared to the classification of results based on combined data from the membership index. Fifteen out of 25 genotypes placed within the same category obtained from the combined data from the membership index and clustering analysis. HT showed deviation in two genotypes (Gemmeiza-9 and DHL05) to group T, and five genotypes deviation from group S to group HS, which was absent in the combined data from the membership index. Nevertheless, according to the Mantel test, the relationship between membership index and clustering of drought-tolerant and sensitive genotypes was significantly correlated (r = 0.328, *p* < 0.0001, alpha = 0.05).

Furthermore, PCoA revealed compatible relationships with cluster analysis of these genotypes, which showed that the genotypes’ stability in their classification groups (Figure 3). This is consistent with the ANOVA results, which revealed highly significant differences for treatments and their interactions (genotypes, irrigation and seasons), thus indicating the high genetic diversity between genotypes. PCoA can be an effective and necessary method for separation of genotypes when clearly different from other genotypes [71,72]. The use of a membership index and clustering analysis were confirmed using discriminant analysis in order to increase classification reliability for water-deficit stress tolerance. The results showed that the contributions were robust, as indicated by MANOVA and discriminant functions, and there were clear separations between the water-deficit tolerance groups. The findings also showed that Can1 was a measure of the overall characteristics of drought tolerance by the four traits, Can2 differentiated genotypes based on it GLA and RWC, and Can3 differentiated genotypes based on GLA and LAI. MANOVA showed that the five groups were significantly different, which indicates complete separation between the five groups based on the four quantitative traits in each S1 and S2. HT vs. T in the GLA trait in S2, I vs. S in the RWC trait in S2, and S vs. HS in the RWC trait in S1, had the same drought tolerance responses (Table S5, Figure 5). HT and T had considerably higher values in the four traits in each S1 and S2, compared to S and HS.

## 5. Conclusions

Eleven interpretive traits to yield combined high heritability (*h*^2^ > 60%) and genetic gain (>20%), compared to GY, which had moderate values of both heritability (57.60%) and genetic gain (16.89%). Selection of interpretive traits could be a substitute for tools for indirect selection of GY under water shortage stress conditions for these reasons. Multidimensional analyses determined four effective interpretive traits (CT, RWC, GLA, and LAI) proposed as comprehensive criteria for selecting water shortage-tolerant genotypes, and selection based on them might improve genetic gain for GY in environments that are vulnerable to water shortages. Discriminant analysis confirmed the results obtained. There were clear separations between water shortage tolerance groups. MANOVA indicated that there was considerable variation between the five water shortage tolerance groups. The six genotypes (DHL02, DHL30, DHL26, Misr1, Pavone-76 and DHL08) can be recommended as interesting new genetic sources for water shortage-tolerant wheat breeding programs.

## Figures and Tables

**Table 1:** Combined analysis of variance for 28 measured traits of 25 wheat genotypes (G) in two seasons (S) under two irrigation regimes (I).

**Source**	**DF**	**LWC**	**RWC**	**CT**	**LEWT**	**Pn**	**Gs**	**Ci**	**E**	**WUE**	
S	1	302.284 **	68.392*	0.589 **	0.00010 **	37.002 **	0.0047 **	16593.14 **	2.869 **	5.763 **	
rep(S)	4	7.271	15.656	0.121	0	0.098	0.0001	1739.11 **	0.087 *	0.049	
I	1	1095.88 **	3711.21 **	516.17 **	0.00007 **	2.869*	0.1647 **	125277.99 **	30.033 **	30.630 **	
S*I	1	0.301	184.836 **	5.057 **	0.00013 **	0.029	0.0007 **	320.974	0.019	1.109 **	
rep(I*S)	4	2.992	6.914	0.107	0	0.287	0.0005	794.38	0.035	0.018	
G	24	82.303 **	124.998 **	2.985 **	0.00026 **	24.174 **	0.0074 **	4028.503 **	1.400 **	2.856 **	
S*G	24	87.854 **	89.304 **	0.793 **	0.00002 **	0.414	0.0003 **	402.104 *	0.072 **	0.769 **	
I*G	24	19.435 **	20.881 **	1.692 **	0.00003 **	4.500 **	0.0000 **	2244.551 **	0.835 **	1.163 **	
S*I*G	24	23.334 **	38.558 **	1.014 **	0.00002 **	0.443	0.0049 **	212.418	0.052	0.655 **	
Error	192	9.543	12.659	0.078	0	0.627	0.0001	294.977	0.038	0.06	
**Source**	**DF**	**WUEi**	**LS**	**GLN**	**FLA**	**GLA**	**LAI**	**DSW**	**DLW**	**TDW**	**DH**
S	1	3923.432 **	0.108 **	4.844 **	2.519	4633.31 **	11.13 **	1.371 *	0.019 **	1.72 **	8.670 **
rep(S)	4	13.004	0.0008	0.185 **	1.827	85.068 **	0.035	0.518	0.008 *	0.421	0.227
I	1	17840.78 **	0.478 **	5.206 **	701.02 **	45628.4 **	300.42 **	40.85 **	0.710 **	52.35 **	145.60 **
S*I	1	2952.544 **	0.0001	0.113	8.653 **	1757.88 **	16.55 **	0.59	0.008	0.73	0.03
rep(I*S)	4	8.258	0	0.05	1.997	28.21	0.054	0.324	0.011	0.306	3.147
G	24	1089.461 **	0.019 **	1.457 **	190.58 **	2308.83 **	9.163 **	48.01 **	0.537 **	52.62 **	164.83 **
S*G	24	287.937 **	0.002 **	0.487 **	9.922 **	544.106 **	2.863 **	0.023	0	0.026	0.121
I*G	24	649.685 **	0.015 **	0.135 **	16.613 **	205.589 **	1.771 **	2.416 **	0.071 **	2.753 **	2.721 **
S*I*G	24	174.860 **	0.001 **	0.151 **	4.897 **	133.676 **	1.216 **	0.035	0.001	0.041	0.079
Error	192	16.693	0.0004	0.039	1.184	14.539	0.034	0.298	0.004	0.321	0.926
**Source**	**DF**	**DM**	**GFD**	**NSP**	**PH**	**SL**	**NSS**	**NG**	**HW**	**GY**	
S	1	6.750 **	5.880 *	590875.3 **	21.87	137.783 **	173.95 **	367.37 **	2363.38 **	3.272 **	
rep(S)	4	3.307 *	0.807	411.987	17.667	0.126	1.346 **	4.756	5.208	0.13	
I	1	1352.6 **	622.1 **	933645.6 **	2296.3 **	27.731 **	64.255 **	1578.9 **	1700.23 **	92.596 **	
S*I	1	0.27	0.48	58408.65 **	383.07 **	0.431	1.952*	1309.0 **	362.362 **	0.536 **	
rep(I*S)	4	3.277	0.764	849.35	12.719	0.188	0.533	2.252	0.797	0.029	
G	24	150.73 **	38.06 **	53328.17 **	440.15 **	7.170 **	18.001 **	227.47 **	314.603 **	11.965 **	
S*G	24	0.076	0.144	18636.06 **	83.814 **	7.029 **	13.620 **	45.081 **	53.422 **	0.713 **	
I*G	24	5.501 **	5.844 **	10219.66 **	33.791 **	0.861 **	0.597	38.455 **	29.064 **	1.714 **	
S*I*G	24	0.194	0.341	10869.40 **	20.360 **	0.394 **	1.348 **	41.090 **	20.545 **	0.302 **	
Error	192	1.341	1.692	680.423	10.701	0.196	0.379	4.409	4.39	0.078	

* and ** indicate significance at *p* < 0.05 and 0.01, respectively, Leaf water content (LWC), relative water content (RWC), canopy temperature (CT), leaf equivalent water thickness (LEWT), photosynthesis rate (Pn), stomatal conductance (Gs), intracellular CO_2_ concentration (Ci), transpiration rate (E), instantaneous water use efficiency (WUE), intrinsic water use efficiency (WUEi), stomatal limitation value (Ls), green leaf number (GLN), flag leaf area (FLA), and green leaf area (GLA), leaf area index (LAI), dry stem weight (DSW), dry leaf weight (DLW), total dry weight (TDW), days to heading (DH), days to maturity (DM), grain filling duration (GFD), plant height (PH), spike length (SL), number of spikes (NS), number of spikelets (NSS), number of kernels (NKS), thousand-kernel weight (HKW), and grain yield (GY).

**Table 2:** Means± standard deviation, ranges of the 25 genotypes of two seasons and their combined for 28 traits.

Traits	Seasons	Full			Limited			Combined Data		
		Min	Max	Mean	Min	Max	Mean	Min	Max	Mean
LWC	Season1	69.18	85.52	74.48 ± 4.02 **	61.39	79.27	70.66 ± 3.52 **	68.89	78.69	73.55 ± 2.08 **
	Season2	68.59	82.62	76.44 ± 3.31 **	67.42	76.12	72.64 ± 2.29 **			
RWC	Season1	77.35	89.42	84.18 ± 3.03 **	69.96	83.01	78.83 ± 3.13 **	73.11	85.62	81.01 ± 2.53 **
	Season2	71.34	91.71	84.82 ± 4.76 **	68.44	81.78	76.21 ± 3.98 **			
CT	Season1	16.29	18.58	17.38 ± 0.55 **	18.88	20.40	19.73 ± 0.43 **	17.78	19.08	18.51 ± 0.42 **
	Season2	15.47	18.25	17.02 ± 0.79 **	18.75	21.17	19.91 ± 0.61 **			
LEWT	Season1	0.011	0.028	0.025 ± 0.00 **	0.013	0.025	0.024 ± 0.00 **	0.011	0.025	0.022 ± 0.00 **
	Season2	0.010	0.026	0.024 ± 0.00 **	0.011	0.021	0.018 ± 0.00 **			
Pn	Season1	8.67	12.93	11.39 ± 1.00 **	8.24	13.27	11.17 ± 1.37 **	8.44	12.49	10.93 ± 1.05 **
	Season2	8.24	11.97	10.67 ± 1.00 **	8.15	12.84	10.49 ± 1.35 **			
Gs	Season1	0.15	0.24	0.19 ± 0.03 **	0.09	0.18	0.14 ± 0.02 **	0.13	0.20	0.16 ± 0.02 **
	Season2	0.12	0.24	0.18 ± 0.03 **	0.08	0.18	0.13 ± 0.02 **			
Ci	Season1	281.51	378.75	330.98 ± 19.35 **	247.41	325.01	287.9 ± 21.3 **	276.80	331.33	302.0 ± 13.4 **
	Season2	284.10	343.27	314.03 ± 13.16**	228.71	305.86	275.1 ± 19.9**			
E	Season1	2.55	3.95	3.27 ± 0.39 **	1.96	3.30	2.62 ± 0.28 **	2.31	3.52	2.85 ± 0.27 **
	Season2	2.26	3.79	3.07 ± 0.36 **	1.75	3.13	2.45 ± 0.34 **			
WUE	Season1	2.90	4.50	3.57 ± 0.41 **	3.44	5.47	4.33 ± 0.51 **	3.12	4.58	3.81 ± 0.35 **
	Season2	2.67	4.56	3.41 ± 0.44 **	2.50	4.75	3.94 ± 0.59 **			
WUEi	Season1	50.53	78.66	61.92 ± 8.11 **	63.87	107.11	83.8 ± 12.60 **	59.29	81.23	69.21 ± 6.67 **
	Season2	46.83	85.33	61.04 ± 9.95 **	55.56	86.80	70.10 ± 8.51 **			
Ls	Season1	0.19	0.36	0.26 ± 0.04 **	0.26	0.48	0.34 ± 0.05 **	0.28	0.39	0.32 ± 0.03 **
	Season2	0.22	0.39	0.30 ± 0.04 **	0.29	0.45	0.38 ± 0.04 **			
GLN	Season1	32.07	45.67	38.53 ± 3.69 **	18.03	44.00	29.78 ± 5.04 **	29.71	44.82	35.27 ± 3.27 **
	Season2	30.65	43.94	36.57 ± 3.34 **	26.84	45.92	36.20 ± 4.12 **			
FLA	Season1	17.82	33.06	23.54 ± 3.50 **	15.93	25.42	20.14 ± 2.34 **	16.39	29.36	21.76 ± 2.91 **
	Season2	16.17	32.73	23.03 ± 3.72 **	14.09	27.61	20.35 ± 3.05 **			
GLA	Season1	66.00	113.83	88.14 ± 12.22 **	40.06	83.98	58.52 ± 10.81 **	54.36	102.68	77.24 ± 10.72 **
	Season2	56.75	131.18	91.08 ± 15.64 **	47.16	96.10	71.22 ± 10.96 **			
LAI	Season1	2.45	5.40	3.80 ± 0.94 **	1.17	3.10	2.12 ± 0.64 **	2.13	3.65	2.96 ± 0.60 **
	Season2	2.11	5.11	3.45 ± 0.83 **	1.29	3.23	2.30 ± 0.49 **			
DSW	Season1	6.18	11.37	8.44 ± 1.37 **	5.48	9.57	7.62 ± 1.16 **	6.01	10.41	7.97 ± 1.22 **
	Season2	6.18	10.95	8.23 ± 1.31 **	5.27	9.74	7.58 ± 1.24 **			
DLW	Season1	0.64	1.34	1.01 ± 0.20 **	0.70	1.16	0.90 ± 0.14 **	0.67	1.23	0.95 ± 0.16 **
	Season2	0.64	1.32	0.99 ± 0.19 **	0.69	1.20	0.90 ± 0.15 **			
TDW	Season1	6.82	12.49	9.46 ± 1.45 **	6.26	10.73	8.52 ± 1.24 **	6.68	11.55	8.92 ± 1.30 **
	Season2	6.82	12.03	9.21 ± 1.39 **	6.03	10.95	8.48 ± 1.32 **			
DH	Season1	71.00	80.33	75.40 ± 2.73 **	70.00	78.33	74.04 ± 2.64 **	70.67	79.50	74.88 ± 2.67 **
	Season2	71.33	80.67	75.71 ± 2.79 **	70.33	78.67	74.39 ± 2.63 **			
MD	Season1	118.33	127.00	121.61 ± 2.71 **	114.67	121.67	117.28 ± 2.16 **	117.00	123.83	119.59 ± 2.36 **
	Season2	118.67	126.33	121.84 ± 2.69 **	115.33	122.00	117.63 ± 2.12 **			
GFD	Season1	42.67	49.67	45.95 ± 1.52 **	40.00	45.33	43.00 ± 1.60 **	41.50	47.50	44.61 ± 1.41 **
	Season2	43.00	50.00	46.13 ± 1.47 **	40.33	45.67	43.35 ± 1.55 **			
NSP	Season1	433.33	770.00	579.07 ± 93.35 **	349.67	570.00	440.39 ± 54.31 **	384.08	544.17	465.55 ± 43.54 **
	Season2	393.33	573.33	462.93 ± 48.38 **	286.67	496.67	379.80 ± 44.76 **			
PH	Season1	73.00	97.00	83.45 ± 7.04 **	67.92	84.67	75.56 ± 4.46 **	71.71	87.47	79.25 ± 4.75 **
	Season2	71.33	91.00	80.67 ± 5.34 **	66.67	85.67	77.31 ± 5.21 **			
SL	Season1	8.73	11.27	10.06 ± 0.75 **	8.17	10.83	9.38 ± 0.70 **	8.01	9.84	9.04 ± 0.50 **
	Season2	6.67	10.60	8.63 ± 0.93 **	6.33	9.53	8.10 ± 0.84 **			
NSS	Season1	13.60	17.56	16.01 ± 1.06 **	13.61	16.83	15.25 ± 0.85 **	13.09	16.32	14.88 ± 0.94 **
	Season2	11.67	17.11	14.66 ± 1.56 **	10.80	15.90	13.59 ± 1.40 **			
NG	Season1	32.07	45.67	38.53 ± 3.69 **	18.03	44.00	29.78 ± 5.04 **	29.71	44.82	35.27 ± 3.27 **
	Season2	30.65	43.94	36.57 ± 3.34 **	26.84	45.92	36.20 ± 4.12 **			
HW	Season1	42.32	61.97	51.72 ± 5.26 **	35.81	52.38	44.88 ± 4.70 **	37.24	52.68	45.53 ± 3.97 **
	Season2	32.89	50.57	44.01 ± 4.45 **	32.72	46.91	41.51 ± 3.98 **			
GY	Season1	3.71	7.24	5.15 ± 0.86 **	2.90	5.99	3.99 ± 0.68 **	3.50	6.49	4.68 ± 0.66 **
	Season2	3.80	7.19	5.31 ± 0.83 **	3.27	6.20	4.25 ± 0.61 **			

** indicate significance at *p* < 0.01, Leaf water content (LWC), relative water content (RWC), canopy temperature (CT), leaf equivalent water thickness (LEWT), photosynthesis rate (Pn), stomatal conductance (Gs), intracellular CO_2_ concentration (Ci), transpiration rate (E), instantaneous water use efficiency (WUE), intrinsic water use efficiency (WUEi), stomatal limitation value (Ls), green leaf number (GLN), flag leaf area (FLA), and green leaf area (GLA), leaf area index (LAI), dry stem weight (DSW), dry leaf weight (DLW), total dry weight (TDW), days to heading (DH), days to maturity (DM), grain filling duration (GFD), plant height (PH), spike length (SL), number of spikes (NS), number of spikelets (NSS), number of kernels (NKS), thousand-kernel weight (HKW), and grain yield (GY).

**Table 3:** Estimates of heritability (*h*^2^), genotypic coefficient of variance (GCV), phenotypic coefficient (PCV), genetic advance (GA), and genetic gain (GG) for 28 measured traits of 25 wheat genotypes in two seasons under two irrigation regimes genotypes.

Traits	*h* ^2^	GCV	PCV	GA	GG
LWC	36.06	2.76	4.60	2.51	3.14
RWC	86.36	10.73	11.55	16.64	20.55
CT	88.39	10.33	10.99	3.70	20.01
LEWT	88.30	26.71	28.42	0.01	51.71
Pn	80.81	11.73	13.05	2.37	21.73
Gs	30.37	8.53	15.48	0.02	9.69
Ci	38.81	3.82	6.13	14.79	4.90
E	38.92	7.48	11.99	0.27	9.61
WUE	55.30	9.52	12.80	0.56	14.58
WUEi	29.99	7.54	13.78	5.89	8.51
Ls	41.37	9.80	15.24	0.04	12.99
GLN	66.92	7.40	9.05	0.48	12.47
FLA	88.65	17.23	18.30	7.28	33.42
GLA	73.32	15.39	17.97	20.95	27.14
LAI	62.70	18.90	23.87	1.13	30.83
DSW	94.46	24.43	25.13	3.90	48.91
DLW	86.26	20.72	22.31	0.38	39.65
TDW	94.27	22.83	23.51	4.08	45.66
DH	97.82	4.91	4.96	7.49	10.00
MD	95.63	2.91	2.98	7.01	5.86
GFD	81.86	3.69	4.08	3.06	6.87
NSP	65.48	11.65	14.39	90.46	19.41
PH	77.91	6.75	7.65	9.72	12.27
SL	39.48	7.06	11.23	0.82	9.14
NSS	27.37	4.39	8.40	0.70	4.74
NG	80.41	11.12	12.40	7.25	20.55
HW	80.31	10.08	11.24	8.47	18.60
GY	57.60	10.80	14.24	0.79	16.89

Leaf water content (LWC), relative water content (RWC), canopy temperature (CT), leaf equivalent water thickness (LEWT), photosynthesis rate (Pn), stomatal conductance (Gs), intracellular CO_2_ concentration (Ci), transpiration rate (E), instantaneous water use efficiency (WUE), intrinsic water use efficiency (WUEi), stomatal limitation value (Ls), green leaf number (GLN), flag leaf area (FLA), and green leaf area (GLA), leaf area index (LAI), dry stem weight (DSW), dry leaf weight (DLW), total dry weight (TDW), days to heading (DH), days to maturity (DM), grain filling duration (GFD), plant height (PH), spike length (SL), number of spikes (NS), number of spikelets (NSS), number of kernels (NKS), thousand-kernel weight (HKW), and grain yield (GY).

**Table 4:** Multicollinearity diagnosis (tolerance and variance inflation factor) of Pearson product-moment correlation matrix for 28 measured traits.

Traits	Before Excluding Traits		After Excluding Traits		Traits	Before Excluding Traits		After Excluding Traits	
	Tolerance	VIF	Tolerance	VIF		Tolerance	VIF	Tolerance	VIF
DH	0.036	**28.13**	0.205	4.89	LWC	0.465	2.15	0.465	2.15
DM	0.025	**40.34**	--	--	RWC	0.300	3.33	0.301	3.33
GFD	0.053	**19.02**	0.209	4.77	NG	0.402	2.49	0.417	2.40
DSW	0.000	0.00	0.000	0.00	HW	0.221	4.54	0.221	4.53
DLW	0.000	0.00	0.000	0.00	CT	0.176	5.67	0.178	5.62
TDW	0.000	0.00	0.000	0.00	LEWT	0.463	2.16	0.470	2.13
NSP	0.151	6.61	0.152	6.59	Pn	0.184	5.43	0.186	5.39
PH	0.255	3.92	0.260	3.85	Gs	0.146	6.86	0.159	6.28
NL	0.234	4.28	0.234	4.28	Ci	0.109	9.15	0.111	9.02
LAF	0.130	7.70	0.142	7.07	E	0.100	10.02	0.111	9.03
TLAF	0.129	7.74	0.133	7.52	WUE	0.146	6.83	0.147	6.82
LAI	0.120	8.33	0.122	8.20	WUEi	0.103	9.67	0.104	9.62
SL	0.182	5.51	0.185	5.41	Ls	0.117	8.54	0.127	7.85
NSS	0.233	4.29	0.236	4.23	GY	0.291	3.44	0.311	3.22

Variance Inflation Factor (VIF), values in bold indicate multicollinearity.

**Table 5:** Stepwise regression and path coefficient analyses for grain yield (dependent variable) with four yield-related traits (independent variables) for combined data across the two seasons under full and limited irrigation regimes.

Source	Stepwise Regression				Path Coefficient			
					Partitioning the Correlation			R^2^
	Regression Coefficient	*p* Value *	R^2^ Par.	R^2^ Com.	Direct Effect	Indirect Effect	Correlation Value	Direct Effect
Intercept	16.232	<0.0001						
GLA	0.492	0.010	0.104	0.104	−0.065	0.511	0.446	0.004
LAI	0.373	0.001	0.141	0.245	0.264	0.230	0.493	0.069
RWC	0.772	0.030	0.171	0.416	0.189	0.201	0.390	0.036
CT	−0.497	< 0.0001	0.400	0.816	−0.653	0.020	−0.633	0.426
Gs	0.326	0.040	0.070	0.886	−0.173	0.479	0.306	0.030
Indirect effect								0.321
Total R^2^				0.886				0.886
Residual				0.338				0.338

Coefficient partial determination (R^2^ Par.), cumulative coefficient determination (R^2^ Com.), * means *p* value of coefficient partial determination.

**Table 6:** Membership index score for the 25 wheat genotypes based on four selected traits (green leaf area, leaf area index, relative water content and canopy temperature).

No.	Genotypes	GLA		LAI		RWC		CT		Over All data				Over All Data	
		S1	S2	S1	S2	S1	S2	S1	S2	S1	Class	S2	Class	Combined	Class
1	DHL12	0.785	0.865	0.709	0.786	0.726	0.738	0.820	0.836	0.760	T	0.806	HT	0.783	T
2	DHL02	0.792	0.821	0.779	0.844	0.820	0.792	0.851	0.857	0.811	HT	0.829	HT	0.820	HT
3	DHL25	0.710	0.782	0.642	0.712	0.657	0.668	0.742	0.756	0.688	T	0.730	T	0.709	T
4	DHL30	0.876	0.907	0.862	0.933	0.907	0.876	0.988	0.900	0.908	HT	0.904	HT	0.906	HT
5	DHL07	0.688	0.758	0.621	0.689	0.636	0.647	0.754	0.696	0.675	T	0.697	T	0.686	T
6	DHL26	0.834	0.864	0.820	0.889	0.863	0.834	0.941	0.857	0.865	HT	0.861	HT	0.863	HT
7	Gemmeiza-9	0.850	0.847	0.729	0.809	0.710	0.797	0.885	0.817	0.793	T	0.818	HT	0.805	HT
8	DHL11	0.494	0.532	0.525	0.476	0.528	0.589	0.532	0.471	0.520	I	0.517	I	0.518	I
9	KSU106	0.400	0.406	0.311	0.360	0.392	0.487	0.380	0.372	0.371	S	0.406	I	0.389	S
10	Gemmeiza-12	0.473	0.479	0.379	0.399	0.462	0.567	0.454	0.409	0.442	I	0.464	I	0.453	I
11	DHL01	0.708	0.706	0.640	0.672	0.591	0.664	0.738	0.681	0.669	T	0.681	T	0.675	T
12	DHL14	0.330	0.352	0.274	0.198	0.340	0.343	0.320	0.196	0.316	S	0.272	S	0.294	S
13	DHL29	0.428	0.479	0.361	0.380	0.440	0.540	0.433	0.390	0.415	I	0.447	I	0.431	I
14	DHL15	0.347	0.408	0.303	0.310	0.376	0.379	0.330	0.275	0.339	S	0.343	S	0.341	S
15	DHL06	0.330	0.389	0.274	0.310	0.358	0.361	0.315	0.199	0.319	S	0.315	S	0.317	S
16	Misr1	0.892	0.925	0.878	0.951	0.878	0.937	0.980	0.917	0.907	HT	0.932	HT	0.920	HT
17	DHL05	0.822	0.906	0.743	0.824	0.761	0.773	0.852	0.832	0.794	T	0.834	HT	0.814	HT
18	Giza-168	0.695	0.766	0.628	0.697	0.643	0.654	0.726	0.741	0.673	T	0.714	T	0.694	T
19	DHL23	0.571	0.611	0.573	0.580	0.636	0.641	0.584	0.573	0.591	I	0.601	T	0.596	I
20	Sakha-93	0.372	0.396	0.294	0.332	0.404	0.368	0.321	0.295	0.347	S	0.348	S	0.348	S
21	DHL21	0.323	0.344	0.195	0.288	0.351	0.320	0.279	0.256	0.287	S	0.302	S	0.295	S
22	DHL22	0.347	0.370	0.289	0.295	0.377	0.344	0.300	0.276	0.328	S	0.321	S	0.325	S
23	DHL03	0.800	0.841	0.761	0.723	0.740	0.752	0.815	0.852	0.779	T	0.792	T	0.786	T
24	Pavone-76	0.776	0.804	0.803	0.787	0.803	0.776	0.833	0.839	0.804	HT	0.801	HT	0.803	HT
25	DHL08	0.834	0.864	0.864	0.846	0.863	0.834	0.896	0.903	0.864	HT	0.862	HT	0.863	HT

Green leaf area (GLA), leaf area index (LAI), relative water content (RWC) canopy temperature (CT), highly tolerant (HT), tolerant (T), intermediate (I), sensitive (S) and highly sensitive (HS).

## Data Availability

All data is contained within the article or supplementary material.

## Supplementary Materials

The following are available online at https://www.mdpi.com/article/10.3390/plants10050931/s1, Table S1. Names and pedigree of the 25 bread wheat genotypes (7 cultivars and 18 doubled haploid lines (DHLs)) used in this study; Table S2. Analysis of variance for 28 measured traits of 25 wheat genotypes (G) in two seasons (S) under two irrigation regimes (I); Table S3. Principal component analysis of 25 wheat genotypes, eigenvalues, proportion, and cumulative variance for the first seven components for 27 measured traits of 100 treatments; Table S4. Total canonical structure of eigenvalue, canonical discriminant function and class means of drought group to canonical discriminant function; Table S5. Summary (LS means) of all pairwise comparisons for Class (Duncan).
