# Supplementary material for: Combining Genetic and Multidimensional Analyses to Identify Interpretive Traits Related to Water Shortage Tolerance as an Indirect Selection Tool for Detecting Genotypes of Drought Tolerance in Wheat Breeding
Source: Plants (Basel). 2021 May 7;10(5):931. doi: 10.3390/plants10050931 (PMC8148561; doi:10.3390/plants10050931)
Supplement: Supplementary file 1 [file plants-10-00931-s001.zip › plants-1184499-supplementary.pdf]

**Table S1.** Names and pedigree of the 25 bread wheat genotypes (7 cultivars and 18 doubled haploid lines (DHLs)) used in this study.

| Name | Pedigree |
|---|---|
| Giza-168 | MRL/BUC//SERICM 93046-8 M-OY-OM-2Y-OB-OGZ. |
| Gemmeiza-9 | Ald“s”/Huac//CMH74 .630/SxCGM 4583 -5GM- 1GM- OGM |
| Gemmeiza-12 | OTUS/3/SARA/THB//VEEMSS97Y00227S-5y-010M-010Y-010M-2Y-1M-0Y-OGM |
| Sakha-93 | Sakha 92/TR810328 S8871-IS-2S-IS-0S |
| Misr1 | OASSIS / SKAUZ//4*BCN/3/2*PATOR CMSS00Y01881T-050M-030Y-030M-030WGY-33M-0Y-0S |
| Pavone-76 | Vcm//Cno/7C/3/Kal/Bb |
| KSU106 | Barouk/R1474-75-3-53-3-3 |
| DHLs (21,22,23,25) | Derived from the cross (Line-115 × Gemmeiza-7) (El-Hennawy et al. 2011) |
| DHLs (5,7,8,11) | Derived from the cross (Line-115 × Giza-164) (El-Hennawy et al. 2011) |
| DHLs (12,14,15,26,29, 30) | Derived from the cross (Gemmeiza-7× Giza-164) (El-Hennawy et al. 2011) |
| DHLs (1,2,3,6) | Derived from the cross (Giza-164× Giza-168) (El-Hennawy et al. 2011) |

**Table S2.** Analysis of variance for 28 measured traits of 25 wheat genotypes (G) in two seasons (S) under two irrigation regimes (I).

| Source | DF | | LWC | | | RWC | | | CT | | |
|---|---|---|---|---|---|---|---|---|---|---|---|
| | S | Comb. | S1 | S2 | Comb. | S1 | S2 | Comb. | S1 | S2 | Comb. |
| S | | 1 | | | 302.284** | | | 68.392* | | | 0.589** |
| rep(S) | 2 | 4 | 13.836 | 0.706 | 7.271 | 25.686 | 5.626 | 15.656 | 0.095 | 0.147 | 0.121 |
| I | 1 | 1 | 566.248** | 529.934** | 1095.882** | 1119.79** | 2776.25** | 3711.21** | 209.52** | 311.70** | 516.17** |
| S*I | | 1 | | | 0.301 | | | 184.836** | | | 5.057** |
| rep*I | 2 | 4 | 8.093 | 20.749 | 5.983 | 1.962 | 0.912 | 13.827 | 0.165 | 0.141 | 0.214 |
| G | 24 | 24 | 98.288** | 71.869** | 82.303** | 70.330** | 143.972** | 124.998** | 1.422** | 2.356** | 2.985** |
| S*G | | 24 | | | 87.854** | | | 89.304** | | | 0.793** |
| I*G | 24 | 24 | 27.579** | 15.189** | 19.435** | 18.976** | 40.462** | 20.881** | 0.787** | 1.919** | 1.692** |
| S*I*G | | 24 | | | 23.334** | | | 38.558** | | | 1.014** |
| Error | 96 | 192 | 9.898 | 9.187 | 9.543 | 10.347 | 14.970 | 12.659 | 0.109 | 0.047 | 0.078 |

Table S2. Cont.

| Source | DF | | LEWT | | | Pn | | | Gs | | |
|---|---|---|---|---|---|---|---|---|---|---|---|
| | S | Comb. | S1 | S2 | Comb. | S1 | S2 | Comb. | S1 | S2 | Comb. |
| S | | 1 | | | 0.00010** | | | 37.002** | | | 0.0047** |
| rep(S) | 2 | 4 | 0.000002 | 0.00000 | 0.00000 | 0.006 | 0.191 | 0.098 | 0.0002 | 0.0000 | 0.0001 |
| I | 1 | 1 | 0.000005** | 0.00019** | 0.00007** | 1.737 | 1.162 | 2.869* | 0.0933** | 0.0722** | 0.1647** |
| S*I | | 1 | | | 0.00013** | | | 0.029 | | | 0.0007** |
| rep*I | 2 | 4 | 0.000000 | 0.00000 | 0.00000 | 0.829 | 0.386 | 0.573 | 0.0000 | 0.0000 | 0.0001 |
| G | 24 | 24 | 0.000146** | 0.00014** | 0.00026** | 11.983** | 12.605** | 24.174** | 0.0038** | 0.0039** | 0.0074** |
| S*G | | 24 | | | 0.00002** | | | 0.414 | | | 0.0003** |
| I*G | 24 | 24 | 0.000025** | 0.00002** | 0.00003** | 2.332** | 2.611** | 4.500** | 0.0024** | 0.0026** | 0.0000** |
| S*I*G | | 24 | | | 0.00002** | | | 0.443 | | | 0.0049** |
| Error | 96 | 192 | 0.000001 | 0.00000 | 0.00000 | 0.642 | 0.611 | 0.627 | 0.0001 | 0.0001 | 0.0001 |

Table S2. Cont.

| Source | DF | | Ci | | | E | | | WUE | | |
|---|---|---|---|---|---|---|---|---|---|---|---|
| | S | Comb. | S1 | S2 | Comb. | S1 | S2 | Comb. | S1 | S2 | Comb. |
| S | | 1 | | | 16593.14** | | | 2.869** | | | 5.763** |
| rep(S) | 2 | 4 | 809.603 | 2668.615** | 1739.11** | 0.075 | 0.098 | 0.087* | 0.055 | 0.043 | 0.049 |
| I | 1 | 1 | 69140.700** | 56458.27** | 125277.99** | 15.785** | 14.267** | 30.033** | 21.698** | 10.041** | 30.630** |
| S*I | | 1 | | | 320.974 | | | 0.019 | | | 1.109** |
| rep*I | 2 | 4 | 50.168 | 72.300 | 1588.760 | 0.022 | 0.047 | 0.069 | 0.003 | 0.075 | 0.036 |
| G | 24 | 24 | 2869.005** | 1561.602** | 4028.503** | 0.715** | 0.758** | 1.400** | 1.468** | 2.157** | 2.856** |
| S*G | | 24 | | | 402.104* | | | 0.072** | | | 0.769** |
| I*G | 24 | 24 | 1250.061** | 1206.907** | 2244.551** | 0.486** | 0.401** | 0.835** | 0.883** | 0.936** | 1.163** |
| S*I*G | | 24 | | | 212.418 | | | 0.052 | | | 0.655** |
| Error | 96 | 192 | 310.220 | 279.733 | 294.977 | 0.037 | 0.038 | 0.038 | 0.060 | 0.060 | 0.060 |

Table S2. Cont.

| Source | DF | | WUEi | | | Ls | | | GLN | | |
|---|---|---|---|---|---|---|---|---|---|---|---|
| | S | Comb. | S1 | S2 | Comb. | S1 | S2 | Comb. | S1 | S2 | Comb. |
| S | | 1 | | | 3923.432** | | | 0.1079** | | | 4.844** |
| rep(S) | 2 | 4 | 3.826 | 22.182 | 13.004 | 0.0004 | 0.0012 | 0.0008 | 0.030 | 0.340** | 0.185** |
| I | 1 | 1 | 17654.469** | 3138.862** | 17840.787** | 0.2456** | 0.2321** | 0.4776** | 1.893** | 3.426** | 5.206** |
| S*I | | 1 | | | 2952.544** | | | 0.0001 | | | 0.113 |
| rep*I | 2 | 4 | 2.733 | 28.366 | 16.515 | 0.0007 | 0.0001 | 0.0008 | 0.046 | 0.032 | 0.100 |
| G | 24 | 24 | 778.105** | 599.293** | 1089.461** | 0.0112** | 0.0096** | 0.0185** | 1.147** | 0.797** | 1.457** |
| S*G | | 24 | | | 287.937** | | | 0.0023** | | | 0.487** |
| I*G | 24 | 24 | 548.023** | 276.521** | 649.685** | 0.0095** | 0.0068** | 0.0154** | 0.179** | 0.107** | 0.135** |
| S*I*G | | 24 | | | 174.860** | | | 0.0009** | | | 0.151** |
| Error | 96 | 192 | 16.508 | 16.877 | 16.693 | 0.000 | 0.001 | 0.000 | 0.061 | 0.016 | 0.039 |

Table S2. Cont.

| Source | DF | | FLA | | | GLA | | | LAI | | |
|---|---|---|---|---|---|---|---|---|---|---|---|
| | S | Comb. | S1 | S2 | Comb. | S1 | S2 | Comb. | S1 | S2 | Comb. |
| S | | 1 | | | 2.519 | | | 4633.313** | | | 11.13** |
| rep(S) | 2 | 4 | 2.033 | 1.622 | 1.827 | 35.369* | 134.767** | 85.068** | 0.021 | 0.050 | 0.035 |
| I | 1 | 1 | 432.718** | 276.951** | 701.016** | 32649.13** | 14737.16** | 45628.40** | 229.006** | 87.967** | 300.42** |
| S*I | | 1 | | | 8.653** | | | 1757.888** | | | 16.55** |
| rep*I | 2 | 4 | 2.182 | 2.673 | 3.993 | 2.103 | 56.578 | 56.419 | 0.038 | 0.127 | 0.107 |
| G | 24 | 24 | 86.596** | 113.911** | 190.585** | 1339.196** | 1513.739** | 2308.828** | 6.942** | 5.084** | 9.163** |
| S*G | | 24 | | | 9.922** | | | 544.106** | | | 2.863** |
| I*G | 24 | 24 | 12.127** | 9.383** | 16.613** | 129.302** | 209.964** | 205.589** | 1.231** | 1.756** | 1.771** |
| S*I*G | | 24 | | | 4.897** | | | 133.676** | | | 1.216** |
| Error | 96 | 192 | 1.174 | 1.194 | 1.184 | 11.921 | 17.157 | 14.539 | 0.041 | 0.026 | 0.034 |

Table S2. Cont.

| Source | DF | | DSW | | | DWL | | | TDW | | |
|---|---|---|---|---|---|---|---|---|---|---|---|
| | S | Comb. | S1 | S2 | Comb. | S1 | S2 | Comb. | S1 | S2 | Comb. |
| S | | 1 | | | 1.371* | | | 0.019** | | | 1.718** |
| rep(S) | 2 | 4 | 0.689 | 0.348 | 0.518 | 0.005 | 0.010 | 0.008* | 0.583 | 0.260 | 0.421 |
| I | 1 | 1 | 25.627** | 15.811** | 40.848** | 0.433** | 0.284** | 0.710** | 32.723** | 20.358** | 52.350** |
| S*I | | 1 | | | 0.590 | | | 0.008 | | | 0.730 |
| rep*I | 2 | 4 | 0.139 | 0.217 | 0.648 | 0.017 | 0.010 | 0.021 | 0.229 | 0.252 | 0.612 |
| G | 24 | 24 | 24.520** | 23.512** | 48.008** | 0.272** | 0.265** | 0.537** | 26.901** | 25.745** | 52.620** |
| S*G | | 24 | | | 0.023 | | | 0.000 | | | 0.026 |
| I*G | 24 | 24 | 1.280** | 1.170** | 2.416** | 0.037** | 0.035** | 0.071** | 1.467** | 1.327** | 2.753** |
| S*I*G | | 24 | | | 0.035 | | | 0.001 | | | 0.041 |
| Error | 96 | 192 | 0.270 | 0.325 | 0.298 | 0.003 | 0.004 | 0.004 | 0.292 | 0.349 | 0.321 |

Table S2. Cont.

| Source | DF | | DH | | | DM | | | GFD | | |
|---|---|---|---|---|---|---|---|---|---|---|---|
| | S | Comb. | S1 | S2 | Comb. | S1 | S2 | Comb. | S1 | S2 | Comb. |
| S | | 1 | | | 8.670** | | | 6.750** | | | 5.880* |
| rep(S) | 2 | 4 | 0.427 | 0.027 | 0.227 | 2.407 | 4.207 | 3.307* | 1.207 | 0.407 | 0.807 |
| I | 1 | 1 | 74.907** | 70.727** | 145.603** | 695.527** | 657.307** | 1352.563** | 328.560** | 294.000** | 622.080** |
| S*I | | 1 | | | 0.030 | | | 0.270 | | | 0.480 |
| rep*I | 2 | 4 | 5.787 | 6.907 | 6.293 | 3.207 | 3.847 | 6.553 | 0.380 | 1.620 | 1.527 |
| G | 24 | 24 | 81.087** | 83.868** | 164.834** | 75.649** | 75.154** | 150.727** | 19.709** | 18.490** | 38.056** |
| S*G | | 24 | | | 0.121 | | | 0.076 | | | 0.144 |
| I*G | 24 | 24 | 1.448 | 1.352** | 2.721** | 3.138** | 2.557** | 5.501** | 3.129* | 3.056** | 5.844** |
| S*I*G | | 24 | | | 0.079 | | | 0.194 | | | 0.341 |
| Error | 96 | 192 | 1.030 | 0.821 | 0.926 | 1.432 | 1.249 | 1.341 | 1.800 | 1.583 | 1.692 |

Table S2. Cont.

| Source | DF | | NSP | | | PH | | | SL | | |
|---|---|---|---|---|---|---|---|---|---|---|---|
| | S | Comb. | S1 | S2 | Comb. | S1 | S2 | Comb. | S1 | S2 | Comb. |
| S | | 1 | | | 590875.32** | | | 21.870 | | | 137.78** |
| rep(S) | 2 | 4 | 545.807 | 278.167 | 411.987 | 12.713 | 22.622 | 17.667 | 0.159 | 0.093 | 0.126 |
| I | 1 | 1 | 729550.14** | 262504.17** | 933645.65** | 2277.60** | 401.80** | 2296.33** | 17.54** | 10.62** | 27.731** |
| S*I | | 1 | | | 58408.65** | | | 383.070** | | | 0.431 |
| rep*I | 2 | 4 | 889.940 | 2202.167 | 1698.70 | 23.533 | 68.682 | 25.438 | 0.701 | 0.653 | 0.375 |
| G | 24 | 24 | 54457.79** | 17506.44** | 53328.17** | 278.954** | 245.01** | 440.145** | 6.954** | 7.246** | 7.170** |
| S*G | | 24 | | | 18636.06** | | | 83.814** | | | 7.029** |
| I*G | 24 | 24 | 14722.50** | 6366.56** | 10219.66** | 36.544** | 17.607** | 33.791** | 0.489** | 0.765** | 0.861** |
| S*I*G | | 24 | | | 10869.40** | | | 20.360** | | | 0.394** |
| Error | 96 | 192 | 833.846 | 527.000 | 680.423 | 12.323 | 9.079 | 10.701 | 0.167 | 0.225 | 0.196 |

Table S2. Cont.

| Source | DF | | NSS | | | NG | | |
|---|---|---|---|---|---|---|---|---|
| | S | Comb. | S1 | S2 | Comb. | S1 | S2 | Comb. |
| S | | 1 | | | 173.949** | | | 367.369** |
| rep(S) | 2 | 4 | 1.618** | 1.074 | 1.346** | 2.465 | 7.047 | 4.756 |
| I | 1 | 1 | 21.904** | 44.303** | 64.255** | 2881.603** | 6.320 | 1578.91** |
| S*I | | 1 | | | 1.952* | | | 1309.01** |
| rep*I | 2 | 4 | 3.361 | 1.672 | 1.066 | 13.672 | 16.290 | 4.504 |
| G | 24 | 24 | 10.705** | 20.916** | 18.001** | 108.797** | 163.744** | 227.460** |
| S*G | | 24 | | | 13.620** | | | 45.081** |
| I*G | 24 | 24 | 0.639** | 1.307** | 0.597 | 65.001** | 14.545** | 38.455** |
| S*I*G | | 24 | | | 1.348** | | | 41.090** |
| Error | 96 | 192 | 0.322 | 0.436 | 0.379 | 4.614 | 4.203 | 4.409 |

Table S2. Cont.

| Source | DF | | HW | | | GY | | |
|---|---|---|---|---|---|---|---|---|
| | S | Comb. | S1 | S2 | Comb. | S1 | S2 | Comb. |
| S | | 1 | | | 2363.38** | | | 3.272** |
| rep(S) | 2 | 4 | 5.796 | 4.620 | 5.208 | 0.070 | 0.191 | 0.130 |
| I | 1 | 1 | 1816.212** | 246.375** | 1700.23** | 50.425** | 42.347** | 92.596** |
| S*I | | 1 | | | 362.362** | | | 0.536** |
| rep*I | 2 | 4 | 3.909 | 0.294 | 1.594 | 0.035 | 0.011 | 0.057 |
| G | 24 | 24 | 212.467** | 155.558** | 314.603** | 6.903** | 5.775** | 11.965** |
| S*G | | 24 | | | 53.422** | | | 0.713** |
| I*G | 24 | 24 | 28.195** | 21.414** | 29.064** | 0.839** | 1.177** | 1.714** |
| S*I*G | | 24 | | | 20.545** | | | 0.302** |
| Error | 96 | 192 | 5.508 | 3.285 | 4.390 | 0.066 | 0.095 | 0.080 |

**Table S3.** Principal component analysis of 25 wheat genotypes, eigenvalues, proportion, and cumulative variance for the first seven components for 27 measured traits of 100 treatments.

| Value | PC1 | PC2 | PC3 | PC4 | PC5 | PC6 | PC7 |
|---|---|---|---|---|---|---|---|
| Eigenvalue | 10.04 | 3.10 | 2.93 | 2.21 | 1.49 | 1.32 | 0.94 |
| Variability (%) | 37.18 | 11.48 | 10.84 | 8.17 | 5.53 | 4.89 | 3.49 |
| Cumulative % | 37.18 | 48.66 | 59.50 | 67.67 | 73.20 | 78.09 | 81.58 |
| Component loading* | | | | | | | |
| DH | 0.07 | 0.22 | **0.39** | 0.08 | 0.01 | 0.05 | 0.00 |
| GFD | **0.37** | 0.16 | 0.01 | 0.02 | 0.06 | 0.17 | 0.02 |
| DSW | 0.26 | 0.10 | **0.55** | 0.01 | 0.00 | 0.00 | 0.02 |
| DLW | 0.29 | **0.40** | 0.00 | 0.10 | 0.01 | 0.03 | 0.03 |
| TDW | 0.29 | 0.14 | **0.47** | 0.02 | 0.00 | 0.00 | 0.03 |
| NSP | **0.33** | 0.01 | 0.11 | 0.02 | 0.20 | 0.10 | 0.04 |
| PH | **0.39** | 0.18 | 0.00 | 0.07 | 0.05 | 0.00 | 0.02 |
| GLN | 0.24 | 0.24 | **0.26** | 0.02 | 0.01 | 0.01 | 0.00 |
| FLA | **0.44** | 0.01 | 0.24 | 0.03 | 0.05 | 0.05 | 0.00 |
| GLA | **0.62** | 0.02 | 0.00 | 0.21 | 0.02 | 0.04 | 0.00 |
| LAI | **0.70** | 0.03 | 0.03 | 0.03 | 0.02 | 0.09 | 0.01 |
| SL | 0.21 | 0.14 | 0.00 | **0.35** | 0.00 | 0.00 | 0.00 |
| NSS | **0.25** | 0.20 | 0.05 | 0.18 | 0.02 | 0.04 | 0.06 |
| LWC | 0.23 | 0.18 | 0.05 | 0.03 | 0.06 | 0.00 | 0.08 |
| RWC | **0.57** | 0.01 | 0.00 | 0.00 | 0.00 | 0.00 | 0.08 |
| NG | 0.14 | 0.08 | 0.17 | 0.04 | 0.06 | 0.01 | 0.02 |
| HW | 0.24 | 0.00 | **0.27** | 0.22 | 0.06 | 0.02 | 0.02 |
| CT | **0.70** | 0.02 | 0.01 | 0.01 | 0.00 | 0.04 | 0.00 |
| LEWT | 0.00 | 0.08 | 0.05 | 0.28 | 0.04 | 0.00 | **0.33** |
| Pn | 0.05 | 0.12 | 0.03 | 0.12 | **0.52** | 0.00 | 0.10 |
| Gs | **0.70** | 0.05 | 0.00 | 0.02 | 0.09 | 0.05 | 0.02 |
| Ci | **0.66** | 0.06 | 0.00 | 0.05 | 0.07 | 0.00 | 0.01 |
| E | **0.67** | 0.03 | 0.00 | 0.04 | 0.04 | 0.06 | 0.04 |
| WUE | **0.32** | 0.24 | 0.00 | 0.06 | 0.04 | 0.19 | 0.00 |
| WUEi | **0.44** | 0.23 | 0.00 | 0.08 | 0.00 | 0.12 | 0.00 |
| Ls | **0.61** | 0.08 | 0.02 | 0.08 | 0.04 | 0.05 | 0.00 |
| GY | **0.23** | 0.09 | 0.21 | 0.02 | 0.00 | 0.19 | 0.00 |

* values ≥ 0.30 are presented in bold face and indicates traits important for PC definition; days to heading number (DH),

Table S4. Total canonical structure of eigenvalue, canonical discriminant function and class means of drought group to canonical discriminant function.

| parameters | | Can1 | Can2 | Can3 |
|---|---|---|---|---|
| Eigenvalue | | 21.299 | 3.552 | 1.656 |
| Discrimination (%) | | 79.366 | 13.235 | 6.172 |
| Cumulative % | | 79.366 | 92.601 | 98.773 |
| | | | | |
| Variable | | | | |
| GLA | S1 | 0.83 | 0.33 | -0.31 |
| | S2 | 0.73 | 0.11 | -0.46 |
| LAI | S1 | 0.86 | -0.04 | -0.47 |
| | S2 | 0.74 | -0.29 | -0.26 |
| RWC | S1 | 0.79 | 0.24 | 0.40 |
| | S2 | 0.70 | 0.11 | 0.45 |
| CT | S1 | 0.72 | -0.63 | 0.09 |
| | S2 | 0.73 | -0.61 | 0.02 |
| Drought Group | | | | |
| HS | | -3.49 | -2.03 | 1.12 |
| HT | | 6.67 | -0.35 | -0.06 |
| I | | -5.04 | -0.87 | -3.40 |
| S | | -3.41 | 3.71 | 0.80 |
| T | | 0.15 | 0.61 | -0.25 |

First season (S1), Second season (S2), green leaves area (GLA), leaf area index (LAI), relative water content (LAI), canopy temperature (CT) highly tolerant (HT), tolerant (T), intermediate (I), sensitive (S) and highly sensitive (HS).

Table S5. Summary (LS means) of all pairwise comparisons for Class (Duncan)

| Contrast | GLA (S1) | GLA (S2) | LAI (S1) | LAI (S2) | RWC (S1) | RWC (S2) | CT (S1) | CT (S2) |
|---|---|---|---|---|---|---|---|---|
| HT | 0.834 a | 0.864 a | 0.834 a | 0.875 a | 0.856 a | 0.842 a | 0.915 a | 0.879 a |
| T | 0.757 b | 0.809 a | 0.684 b | 0.739 b | 0.683 b | 0.712 b | 0.791 b | 0.776 b |
| I | 0.533 c | 0.572 b | 0.549 c | 0.528 c | 0.582 c | 0.615 c | 0.558 c | 0.522 c |
| S | 0.434 d | 0.454 c | 0.350 d | 0.380 d | 0.431 d | 0.531 c | 0.422 d | 0.391 d |
| HS | 0.342 e | 0.377 d | 0.272 e | 0.289 e | 0.368 d | 0.353 d | 0.311 e | 0.249 e |
| Pr > F | 0.000 | 0.000 | 0.000 | 0.000 | 0.000 | 0.000 | 0.000 | 0.000 |
| Significant | Yes | Yes | Yes | Yes | Yes | Yes | Yes | Yes |
